# Supplementary material for: Nanoscale lattice dynamics in hexagonal boron nitride moiré superlattices
Source: Nat Commun. 2021 Sep 30;12:5741. doi: 10.1038/s41467-021-26072-7 (PMC8484559; doi:10.1038/s41467-021-26072-7)
Supplement: Supplementary file 1 — Supplementary Information [file 41467_2021_26072_MOESM1_ESM.docx]

**Supplemental Materials to Nanoscale Lattice Dynamics in Hexagonal Boron Nitride Moiré Superlattices**

S. L. Moore^1^, C. J. Ciccarino^2^, D. Halbertal^1^, L. J. McGilly^1^, N. R. Finney^3^, K. Yao^3^, Y. Shao^1^, G. Ni^1^, A. Sternbach^1^, E. J. Telford^1^, B. S. Kim^3^, S. E. Rossi^1^, K. Watanabe^4^*,* T. Taniguchi^4^

A. N. Pasupathy^1^, C. R. Dean^1^, J. Hone^3^, P. J. Schuck^3^, P. Narang^2^, D. N. Basov^1^

*^1^Department of Physics, Columbia University, New York, New York. ^2^School of Engineering and Applied Sciences, Harvard University, Cambridge, Massachusetts. ^3^Department of Mechanical Engineering, Columbia University, New York, New York. ^4^National Institute for Materials Science, 1-1 Namiki, Tsukuba, Japan.*

**Supplementary Note** **1: Monolayer hBN identification and stacking procedure**


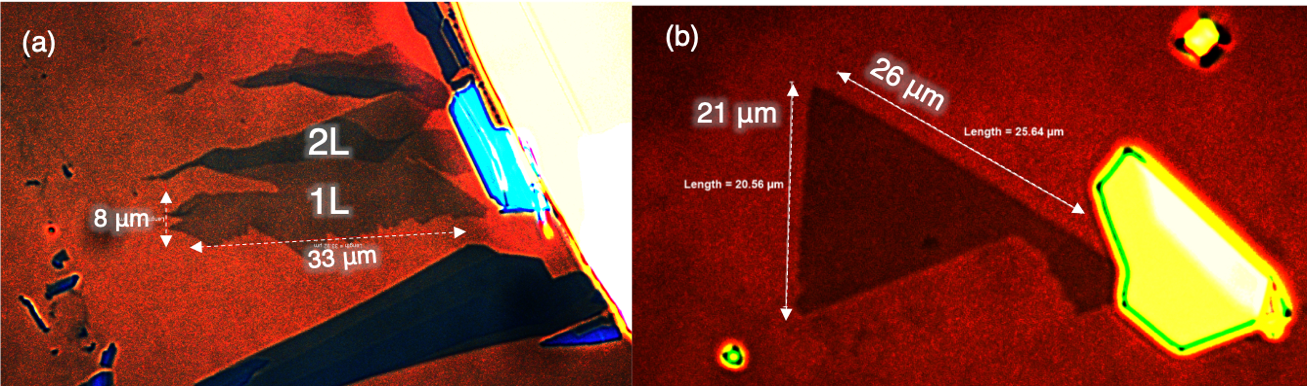


**Supplementary Figure 1****:** **Original flake layouts**. (a) Sample 1, (b) Sample 2. Layer numbers are clarified in (a).

We exfoliate hBN crystals onto 90nm SiO_2_/Si wafers. Monolayers are subsequently identified with an optical microscope using a high-gamma camera setting. Such images are shown for samples 1 and 2 prior to tear-and-stack in Supplementary Figure 1. On occasion, optical contrast identification can confuse between 2L and 1L samples. Moiré-pattern imaging, either with SNOM or PFM, offers direct confirmation of the layer number. The moiré periodicity directly yields the local twist angle. A tear-and-stack procedure without any layer rotation, as with sample 2, should produce triangular domains if the starting flake is a monolayer. If the sample is a natural (AA’) bilayer, this procedure would produce a natural 4-layer sample without the possibility triangular moiré patterns (see Supplementary Note 5 for simulations of AA’ and AA-type moiré patterns). As further confirmation, with sample 1, we produce a triangular network in a 2L/1L arrangement by rotating the sample 60-degrees after tearing off the monolayer in Supplementary Figure 1a. This places the monolayer in AA-type alignment with the top-most layer of the bilayer (as opposed to AA’ alignment).

**Supplementary Note** **2: Additional piezo-force microscopy results**

Piezo-force microscopy (PFM, see methods) enables 5-micron-sized images with ~5 nm spatial resolution in a matter of minutes. The physics behind PFM in t-hBN requires additional details beyond that of twisted bilayer graphene, another system with triangular domain formation^1^. There, the contrast is enhanced along domain walls (SP stacking), where strain gradients are largest, but minimal inside the domains. A contact-mode AFM locks into an AC drive-frequency fixed at a higher-order cantilever resonance. The AFM deflection signal is then demodulated at this drive frequency, which produces a map proportional to the sample polarization. The observed discrepancy between graphene and BN moiré can be understood by recognizing that, unlike bilayer graphene, AA-type hBN breaks inversion symmetry and hosts ferroelectric domains. Quantifying the local piezoelectric coefficient will require separating the artifacts out from single-frequency PFM (used here) with a more advanced mode such as multi-frequency PFM^2^. Despite its qualitative nature, single-frequency PFM is used as a reliable high-throughput characterization tool. Supplementary Figure 2 shows example images on sample 1, while sample 2 was shown in Figure 1 of the main text.

**
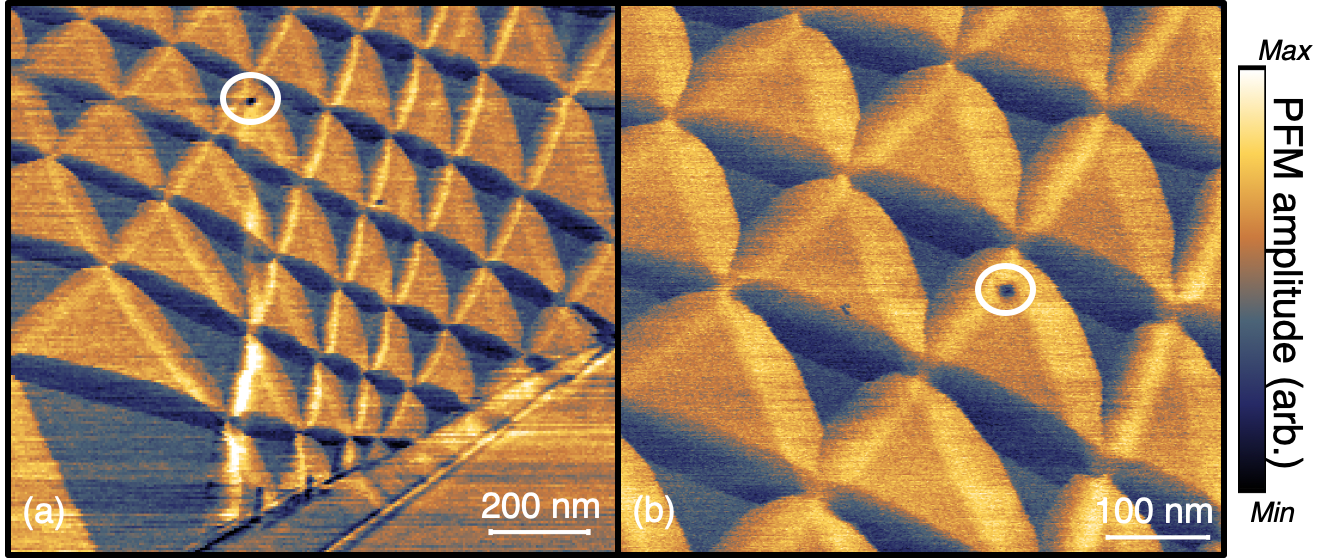
**

**Supplementary Figure 2**: **Single-frequency PFM images of sample 1.** The markers in (a) and (b) correspond to the same region.

**Supplementary Note** **3: Identification of AB/BA domains from dc-EFM**

DC electrical force microscopy (dc-EFM) measurements provide direct evidence for ferroelectricity^3^. Supplementary Figure 3 presents dc-EFM imaging of sample 2 at zero and $\pm$3.14 V bias, applied such that the tip is biased and the sample’s silicon substrate is grounded. Zero bias represents the remnant 2D ferroelectric polarization, where all domain walls appear relatively straight. On the other hand, a finite bias offsets the energy difference between domains, producing pronounced curvature. Since such downward electric fields are expected to create preference for AB stackings^4^, we label the domains accordingly. Such field-induced domain wall motion has been reported previously in studies of twisted multilayer hBN interfaces^5^.


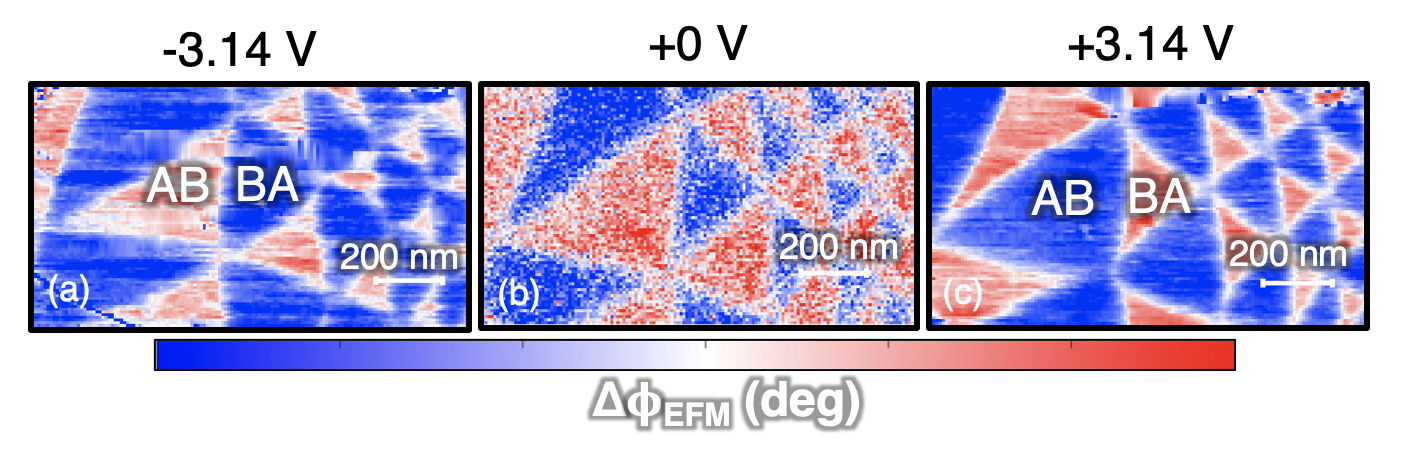


**Supplementary Figure 3****: Sample 2 dc-EFM imaging at various tip bias voltages.**  The reversal in curvature from $V_{tip}=-3.14 V$ (a) to $V_{tip}=3.14 V$(c) allow us to identify the AB and BA domains. The contrast at 0 V (b) is attributed to the Ferroelectric remnant polarization.

**Supplementary Section** **4: Comparison of Topographic and Near-field imaging**

Here we argue that topography is not influencing the observed near-field moiré patterns, and that the response observed in Fig. 1 is purely optical. While Near-field imaging shows pronounced contrast in the moiré-patterned region, the topographic signal shows no contrast. This is not a fundamental expectation: with an AFM of sufficient sensitivity, a moiré pattern can be observed in topography^6^. Typically, topographic height should be largest along domain walls due to relaxation-induced corrugation. In a ferroelectric material like t-hBN, the tip-sample interaction can produce additional topographic responses. The dc-EFM technique then isolates this from standard topographic features. Supplementary Figure 4 compares topography and EFM images, performed without any laser illumination or electrical bias. In this work, none of our topographic images have sufficient fidelity to resolve the moiré, even with the ferroelectric interaction. Ultimately, the best confirmation of the reliability of our near-field imaging is with spectroscopy. As Fig. 1 demonstrates, the contrast is strongly sensitive to the laser frequency, whereas a topographic-induced image would be frequency independent.


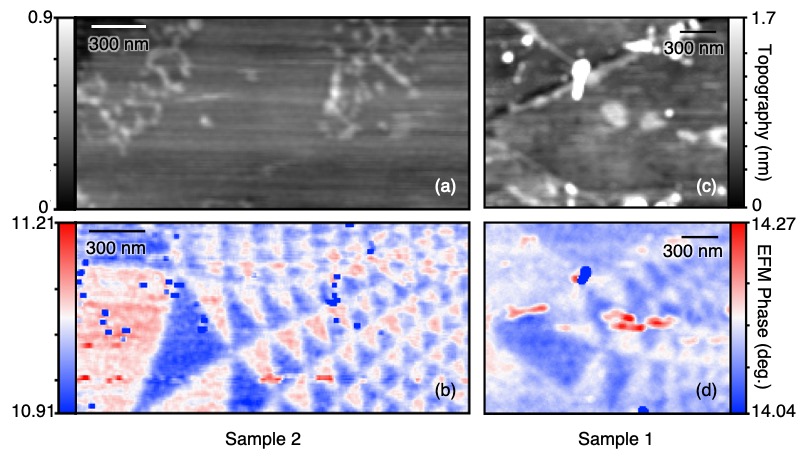


**Supplementary Figure 4: Topography in moiré regions.** Comparison of topography and nano-IR channels for samples 1(c-d) and 2(a-b). Tapping-mode height in (a),(c) shows no observable moiré pattern, while being easily distinguished in the dc-EFM images of (b), (d).

**Supplementary Note** **5: AA and AA’-type moiré relaxation calculations**

Before analyzing the nano-IR data quantitatively, we first checked that the gross features match our expectations from mechanical relaxation calculations. We compare AA and AA’ moiré patterns for two different twist angles (see methods for details). The relaxed stacking energy landscapes are displayed in Supplementary Figure 5. There, we show that AA’-type moiré patterns relax to hexagonal superlattices, consistent with past nano-IR measurements on bulk hBN^7^. Indeed, AA-type moiré shows the experimentally-matching triangular lattice, consistent with our stacking procedures.

We started with previous results of generalized stacking-fault energy (GSFE) calculations^8,9^, which quantify the t-hBN energy at all stacking configurations. There, the Bernal domains were found to be two degenerate points in this configuration space with the lowest energy. Our relaxation calculations combine this stacking energy landscape with monolayer hBN’s mechanical properties^10,11^. In Supplementary Figure 5, we then obtain realistic relaxed moiré maps for two different twist angles: 0.05 (Supplementary Figure 5a and Supplementary Figure 5c) and 1-degrees (Supplementary Figure 5b and Supplementary Figure 5d). The GSFE analysis of hypothetical free-standing t-hBN bilayers confirm that our structure is of the 0.05-degree AA-type relaxed lattice. See Supplementary Note 9 for simulations of the relaxed domains including graphite-modified stacking energies.


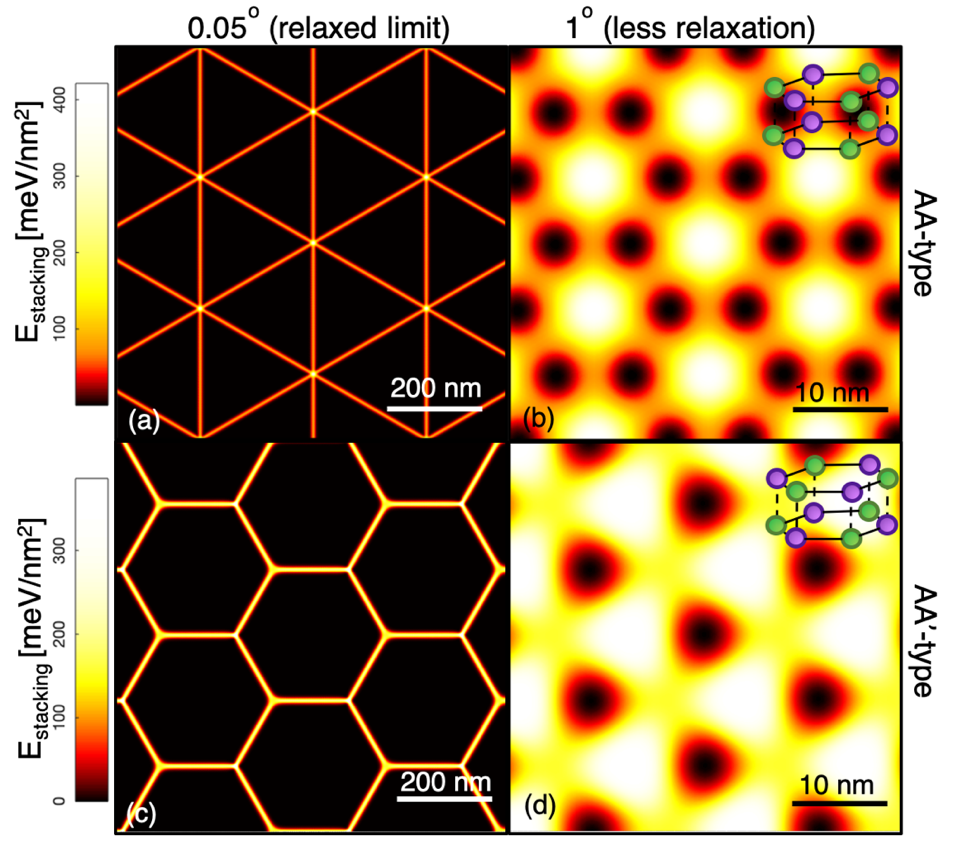


**Supplementary Figure 5:** **Strain relaxation simulations.** Comparison of AA- and AA’-type small-twist-angle moiré patterns. AA-type relaxed-lattice stacking energy with ${0.05}^{o}$ (a) and $1^{o}$ twist angles (b). (c-d) shows AA’-type moiré patterns with ${0.05}^{o}$ and $1^{o}$ twist angles, respectively. The schematic insets in the upper-right corners of panels (b) and (d) show AA and AA’ stackings, respectively.

**Supplementary Note** **6: Additional nano-IR imaging of sample 1**

We now show a selection of sample 1 amplitude and phase images across the phonon resonance. As observed in the main text, minimal phase contrast is typically observed at the frequencies of maximum amplitude contrast, and vice versa.


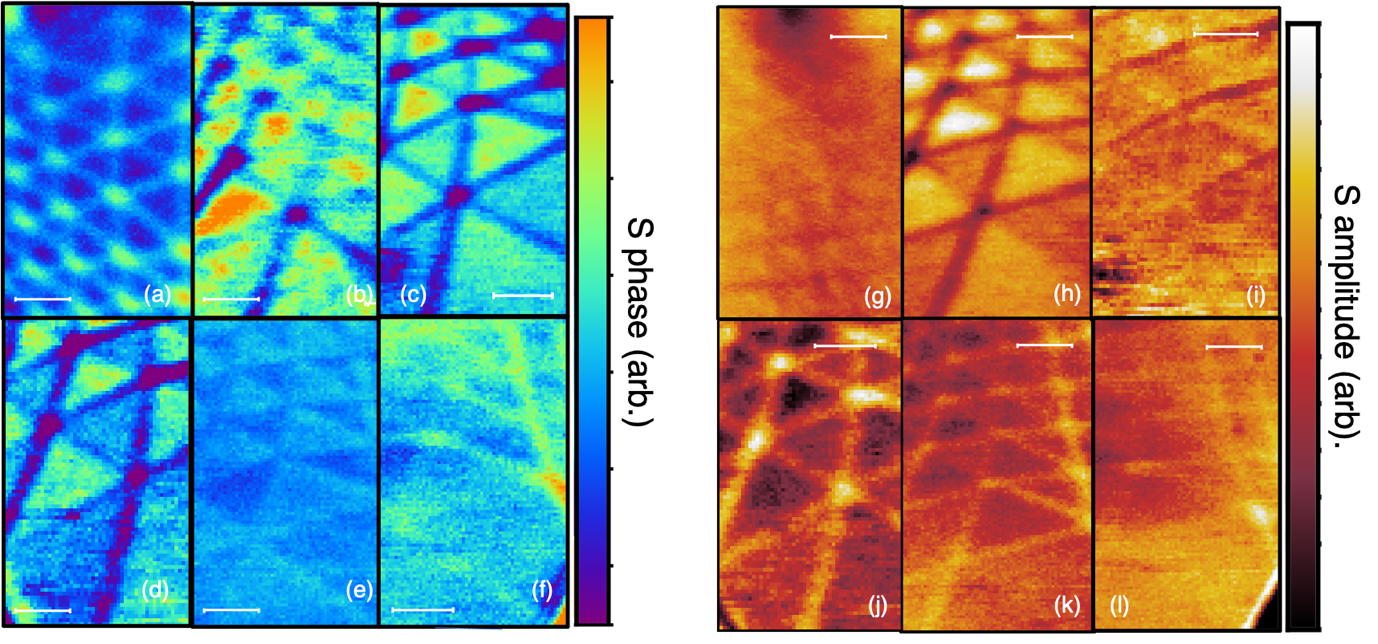


**Supplementary Figure 6: Additional Sample 1 nano-IR imaging results.**  A selection of near-field amplitude and phase images, taken at (a,g) 1360.9 cm^-1^, (b,h) 1367.9 cm^-1^ , (c,i) 1371 cm^-1^, (d,j) 1373 cm^-1^, (e,k) 1376.4 cm^-1^ and (f,l) 1380.4 cm^-1^. Scale bars are all 200 nm.


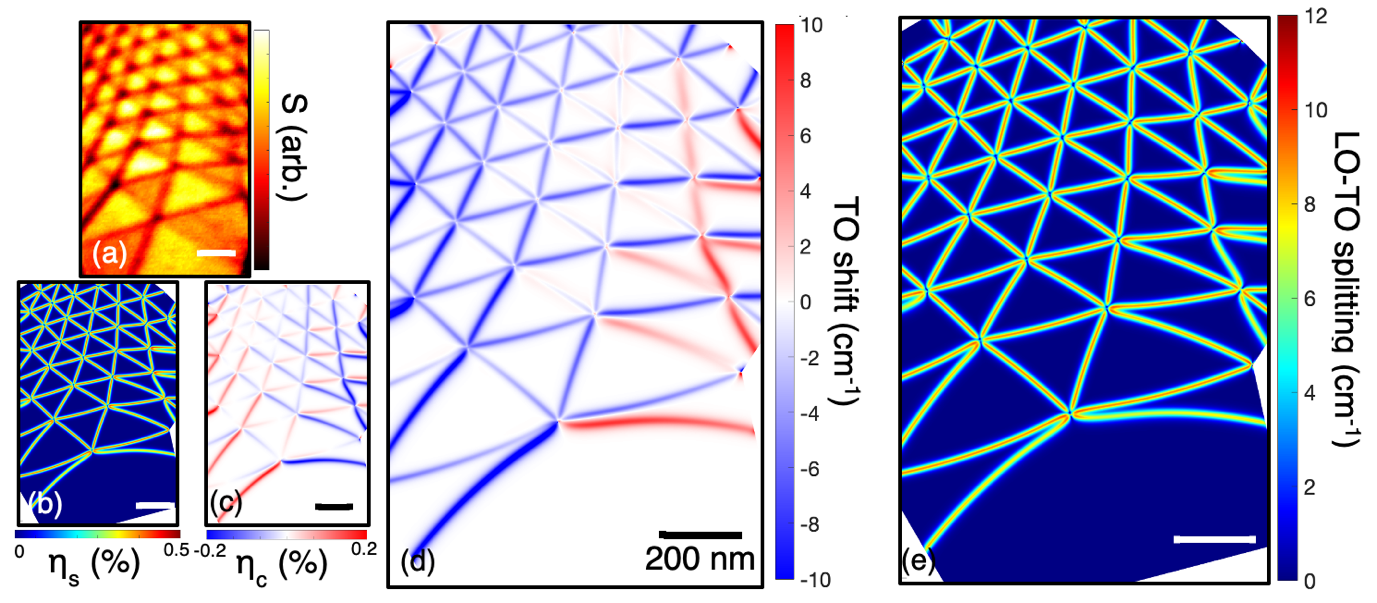


**Supplementary Figure 7: Strain-induced frequency shifts of Sample 1.** Maps of relaxation-induced frequency shifts. (a) near-field amplitude data taken for sample 1 at 1367.9 cm^-1^. Corresponding incompressive (b) and compressive (c) components of the relaxation-induced strain tensor computed through a relaxation simulation (see methods). Simulated (d) TO and (e) LO frequency-shift maps of the same region (derived from b-c and Equation 2).

Next we analyze phonon frequency shifts between domain wall and AA-vortices in Fig. 2. The larger AA and SP-stacking frequency shifts documented in Fig. 2i admit an interpretation in terms of relaxation-induced strain. Using our *ab* stacking energies in Fig. 3b, we evaluated such a strain profile. The only required inputs are the coordinates of AA sites^12^. We define compressive $\eta_{c}$ and shear $\eta_{s}$ strain in terms of the strain tensor $\eta_{ij}$ as:

$\eta_{c}=\frac{\eta_{xx}+\eta_{yy}}{2}; \eta_{s}=\sqrt{\frac{\left( \eta_{xx}-\eta_{yy} \right)^{2}}{4}+\eta_{xy}^{2}}.$(S1)

We then calibrate the strain with the fractional TO-frequency shift via the well-established formula^13^

$\frac{\Delta\omega}{\omega_{TO,i}^{0}}=C\eta_{c}\pm D\eta_{s},$(S2)

where $C$ and $D$ are constants and $\omega_{TO,i}^{0}$ is the unperturbed TO frequency in stacking configuration $i$. Comparing this formula to our ab-initio calculations of strained AA-type BN frequency shifts, we find $C=-0.041 \left( \% \mathrm{strain} \right)^{-1}$ and $D=0.0084 \left( \% \mathrm{strain} \right)^{-1}$ (see methods of main text). Note that in addition to a frequency shift, the $D$ term predicts a splitting into two orthogonal modes. The high-frequency mode has LO symmetry, while the low-frequency mode contributes to the TO-frequency shift, specifically $\Delta\omega_{TO}=\left( C\eta_{c}-D\eta_{s} \right)\omega_{TO,i}^{0}, \omega_{LO}-\omega_{TO}=2D\eta_{s}\omega_{TO,i}^{0}$. This allows us to plot the relaxation-induced strain map for sample 1 and consequently the predicted TO and LO shifts in Supplementary Figure 7.

We see from the maps in Supplementary Figure 7 that, within our model of the substrate-modified AB/BA stacking energies, the SP domain wall and AA-site surrounding areas are the only strained regions. Strain maps also identify a noticeable domain wall curvature. The magnitude of domain wall curvature depends on the hBN/graphite stacking and strain distribution between the hBN layers (see Supplementary Figure 13 and Supplementary Note 9 for more discussion on the latter). The shear strain (Supplementary Figure 7b), providing the LO-TO splitting (Supplementary Figure 7e), shows a repeating profile across all domain walls. The compressive strain profile (Supplementary Figure 7c), providing the TO shift (Supplementary Figure 7d), seems to vary and even switch sign between different domain walls. The smallest twist-angle regions (largest moiré periodicity) show the largest strain. This qualitatively resembles the inhomogeneous domain wall nano-IR response, also displayed in Supplementary Figure 7a. The simulation appears to overestimate this effect, however, partly due to numerical inaccuracies near terminating domain walls and the assumed strain distribution between the layers. However, the remaining inconsistencies in the interior require more systematic investigations of individual domain responses. Meanwhile, Supplementary Figure 7e predicts enhanced LO-TO splitting along the domain walls, which correctly manifests as an increased oscillator strength seen in Fig. 2i. Alterative scenarios invoking, for example, a variation of interlayer spacings are not consistent with our data (see Supplementary Figure 10, Supplementary Figure 11). We therefore conclude that moiré-induced strain plays a central role in the AA and SP frequency shifts, oscillator strengths, and inhomogeneous broadening.

**Supplementary Note** **7: Sample 2 near-field spectroscopy**

Here, we provide further information for the sample 2 data set, corresponding to a monolayer hBN on monolayer hBN configuration. We turn to spectroscopy, as was shown for sample 1 in the main text. The results are seen in Supplementary Figure 8. The overall contrast remains qualitatively the same. We indeed see the same overall trend in TO frequencies as we do in sample 1 (BA the largest, AA the smallest). BA again has the smallest damping, and here AA has the largest damping. The oscillator strength is largest in the AA, SP stackings as before, likely due to the strain-induced LO-TO splitting. However, the quantitative fitting also reveals a blue shift to the TO frequencies by about 1 cm^-1^ compared to sample 1. Small discrepancies between absolute frequencies of different data sets are not as reliable as the 1 cm^-1^ shift found from fitting neighboring AB/BA domains on the same sample. There is nonetheless precedence for monolayers showing bluer phonon frequencies than AA’ bilayers^14,^^15^; the AA’ bilayer on top of sample 1 may soften the apparent TO frequency in the moiré region.


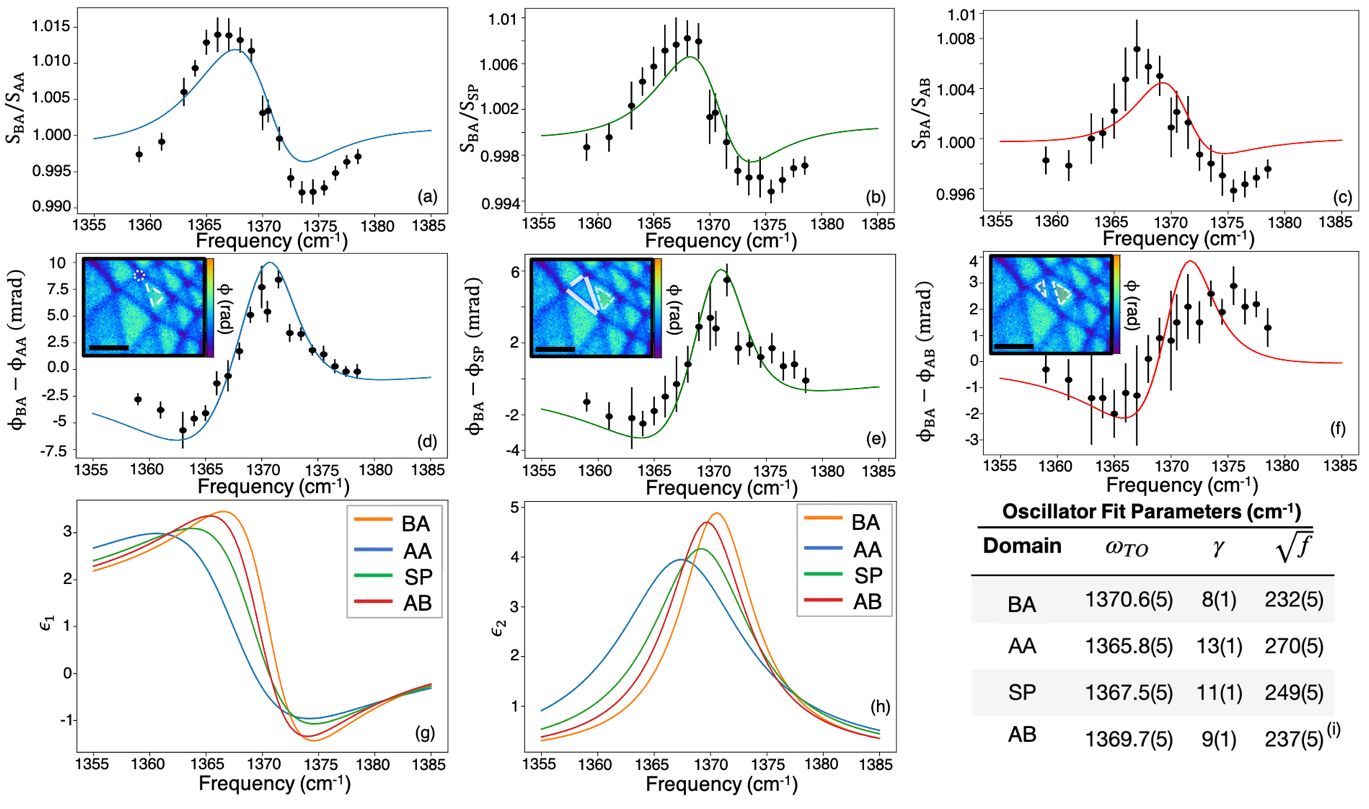


**Supplementary Figure 8: Sample 2 nano-IR spectroscopy.** (a-c) Sample 2 spectra of the scattering amplitude ratios for three independent configurations averaged over AB, BA and SP regions. (d-f) Spectra of the scattering phase differences for three independent configurations averaged over AB, BA and SP regions. The inset is a 1370.5 cm^-1^ phase image marking the regions used to construct spectra in the main panels (scale bar: 200 nm). (g-h) Extracted dielectric function per stacking configuration with (i) extracted phonon parameters, units in wavenumbers. As in Fig. 2, the error bars on the data equal the standard deviation of amplitude and phase responses, while the uncertainties on the fitting parameters are estimated by varying the guess parameters.

The strain maps in Supplementary Figure 9 suggest a similar inhomogeneity in the AA and SP responses as sample 1, which again points to inhomogeneous broadening as a possible source of the increased phonon damping rates.


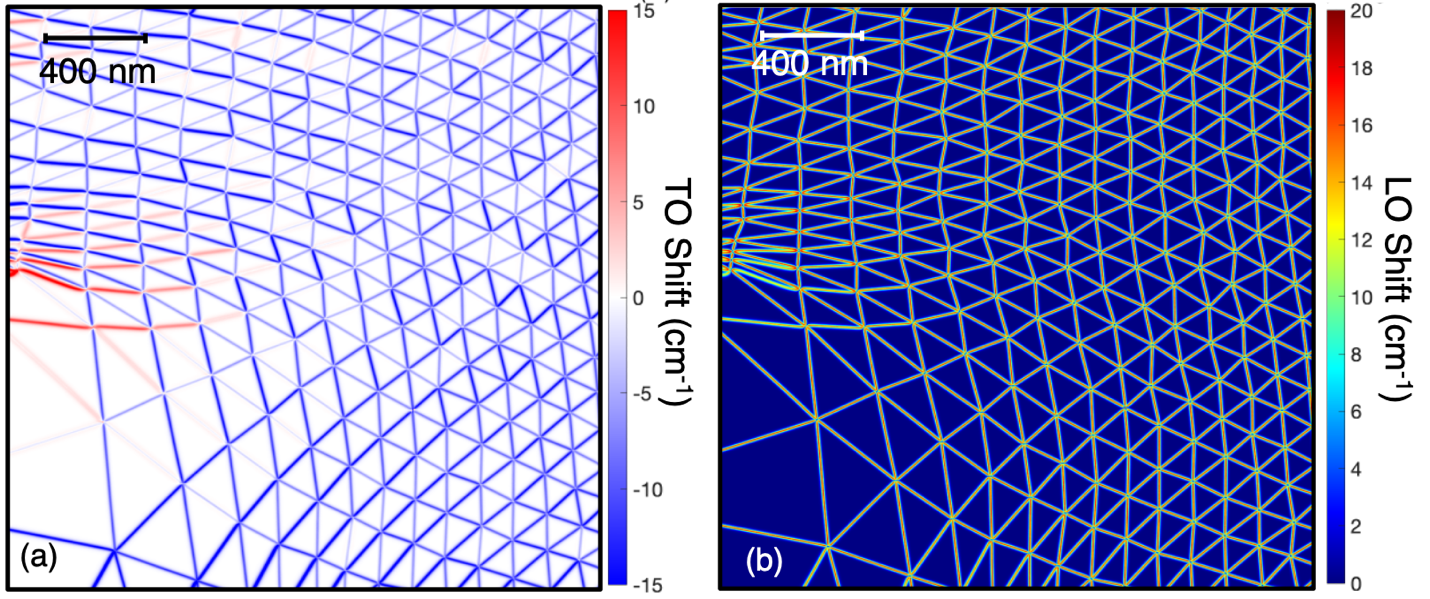


**Supplementary Figure** **9: Strain-induced Frequency shifts of Sample 2.** TO (a) and LO (b) relaxation-induced frequency shifts extracted from the Fig. 1f PFM image of Sample 2.

**Supplementary Note** **8: Role of interlayer spacing on phonon frequencies**

In this section we examine how interlayer spacing affects the TO frequency. Another explanation for the AA and SP frequencies, which has been studied in twisted MoS_2_, is the phonon-frequency dependence on the t-hBN interlayer spacing^16^. Consistent with these studies, such an effect leads to an unreasonably small 0.5 cm^-1^ blue shift of the AA TO frequency relative to the AB TO frequency. Interlayer lattice-constant variation between hBN layers does not agree with our observed 4 cm^-1^ red shift from AB to AA stackings.

The phonon frequencies of bilayer hBN associated with AA and AB stacking are shown in Supplementary Figure 10a as a function of the global van der Waals scaling factor. In our calculations we choose this to be 0.15, however here we include data of similar scaling factors, 0.1 and 0.2. Under these circumstances, we find the TO phonon frequencies associated with AA- and AB-stacked bilayer hBN to be very similar, with the AA frequencies higher in frequency by roughly 0.2 cm^-1^. We note that the AA and AB configurations were found to relax to slightly different in-plane lattice constants of 2.5040 Å (AA) and 2.5047 Å (AB). This very slight difference also contributes to the frequencies found for two stacking configurations. However, overall, these calculated frequencies of the two stacking configurations are both qualitatively and quantitatively different than the experimental results on the moiré structure, where the AA frequencies were about 4 cm^-1^ lower in frequency compared to the BA regions. This suggests that there is a physical mechanism at play besides the layer orientations in isolation, namely strain. In Supplementary Figure 10b we show the evolution of the effective out-of-plane lattice constant *c* as a function of the van der Waals scaling factor associated with the AB configurations shown in Supplementary Figure 10a. The increased vdW interactions in this case causes a decrease in the interlayer separation distance for the two layers of hBN.

**Supplementary Figure 10: Interlayer-spacing-dependence of the AA/AB frequency shift.** The TO frequency AA/AB difference is plotted for (a) three different vdW scaling factors along with (b) the corresponding interlayer spacing difference.

Next, we examine the interlayer separation between hBN and graphite. We employ the same calculations as above, but with an AB/BA modified GSFE. Here, we find a very slight interlayer-spacing separation difference for *ab* stacking relative to *ba* and *aa* stackings (Supplementary Figure 11). Note that AB and BA stackings have the same separation distance and is therefore not the contributing factor for their frequency shift.


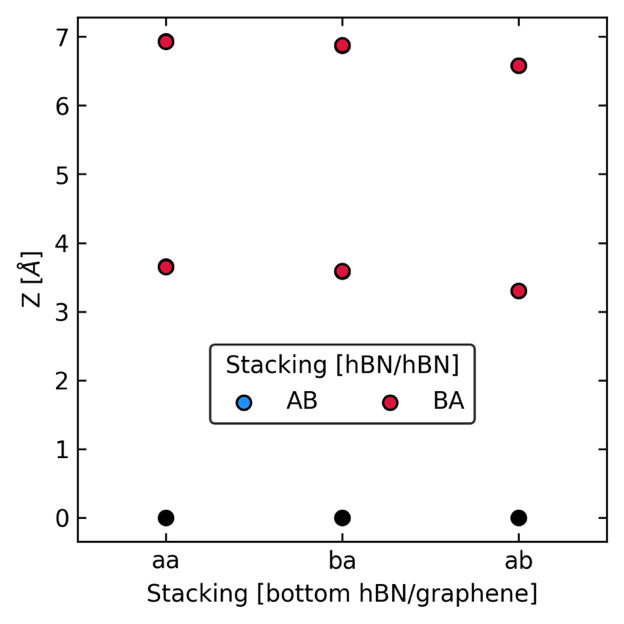


**Supplementary Figure 11:**  **Interlayer-spacing dependence of Bernal t-hBN domains with respect to three hBN/graphene stacking configurations**. The black markers represent the graphene layer, while the blue and red markers represent the BN layers. The blue markers (corresponding to AB stacking) are not visible as they directly overlap with the red markers (corresponding to BA stacking).

Finally, we calculate the dependence of the AB/BA frequency shift on the graphite interlayer separation. Supplementary Figure 12a and Supplementary Figure 12b show that the sensitivity to the commensurate hBN/graphite alignment happens below about 4 Å. Above that point, the stacking energy and frequency shift is finite, decreasing monotonically to zero as the graphite layer separates off to infinity. The squares in Supplementary Figure 12b indicate that the natural separations between hBN and graphite are indeed below this critical value.


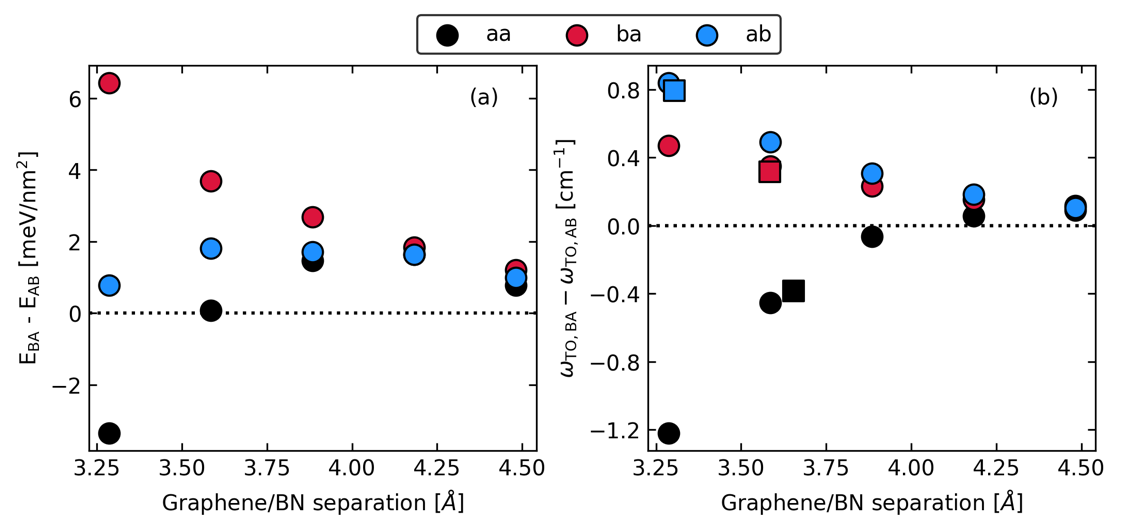


**Supplementary Figure 12:** **Tuning AB/BA phonons with a graphene substrate.** The calculated variation of the BA-AB (a) energy contrast and (b) frequency shifts as a function of bottom BN/graphene separation distance, shown for the three different commensurate graphene stacking configurations presented in Figure 3a. The squares in panel (b) correspond to the frequency shifts for the fully-relaxed configurations, presented in Figure 3c of the main text.

**Supplementary Note** **9: Effect of substrate-modified stacking-energy on relaxation-induced strain profiles**

Here we present relaxation calculations for three different layer arrangements and compare hBN/graphite *ba* and *ab* stacking predictions. The *ba* stacking predicts 5 meV nm^-2^ stacking energy differences, while the *ab* arrangement predicts a value of 2 meV nm^-2^. In addition to this energy difference, the strength of the domain curvature depends on the layer arrangements. The monolayer/monolayer (ML/ML) condition, as in sample 2, can be seen in Supplementary Figure 13a and Supplementary Figure 13d. This predicts larger domain curvature than the bilayer/monolayer condition (sample 1) in Supplementary Figure 13b and Supplementary Figure 13e. Supplementary Figure 13c and Supplementary Figure 13f show even less curvature when assuming the bottom layer is fixed to the substrate and the top layer is only capable of strain relaxation. The relaxation-induced strain distribution between the layers is expected to be uniform, so this latter condition may be less likely. Note that the domain curvature $R$ relates to the measurable domain wall separation $\Delta x$ from the straight domain wall’s midpoint via

$$R=\frac{1}{2\Delta x}\left( \Delta x^{2}+\frac{\lambda_{\mathrm{moire}}^{2}}{4} \right). \left( S3 \right)$$

As expected, larger moiré periods $\lambda_{\mathrm{moire}}$ correspond to greater domain widening. Any experimentally observed domain curvature must satisfy two requirements. First the domain wall displacement $\Delta x$ must be greater than the spatial resolution: 20 nm for s-SNOM, and 5 nm for PFM. Second, the motion must be greater than the domain wall width. Typically the second criterion inhibits curvature resolution in PFM measurements, while the first criterion inhibits spatial resolution in s-SNOM measurements. Due to these issues, the moiré periods in our data are often too small to see significant curvature. However, *ba* stacking should lead to resolvable domain wall separation within our resolution, while *ab* stacking should remain too small. The latter is more consistent with the straight domains we observe in the main text.


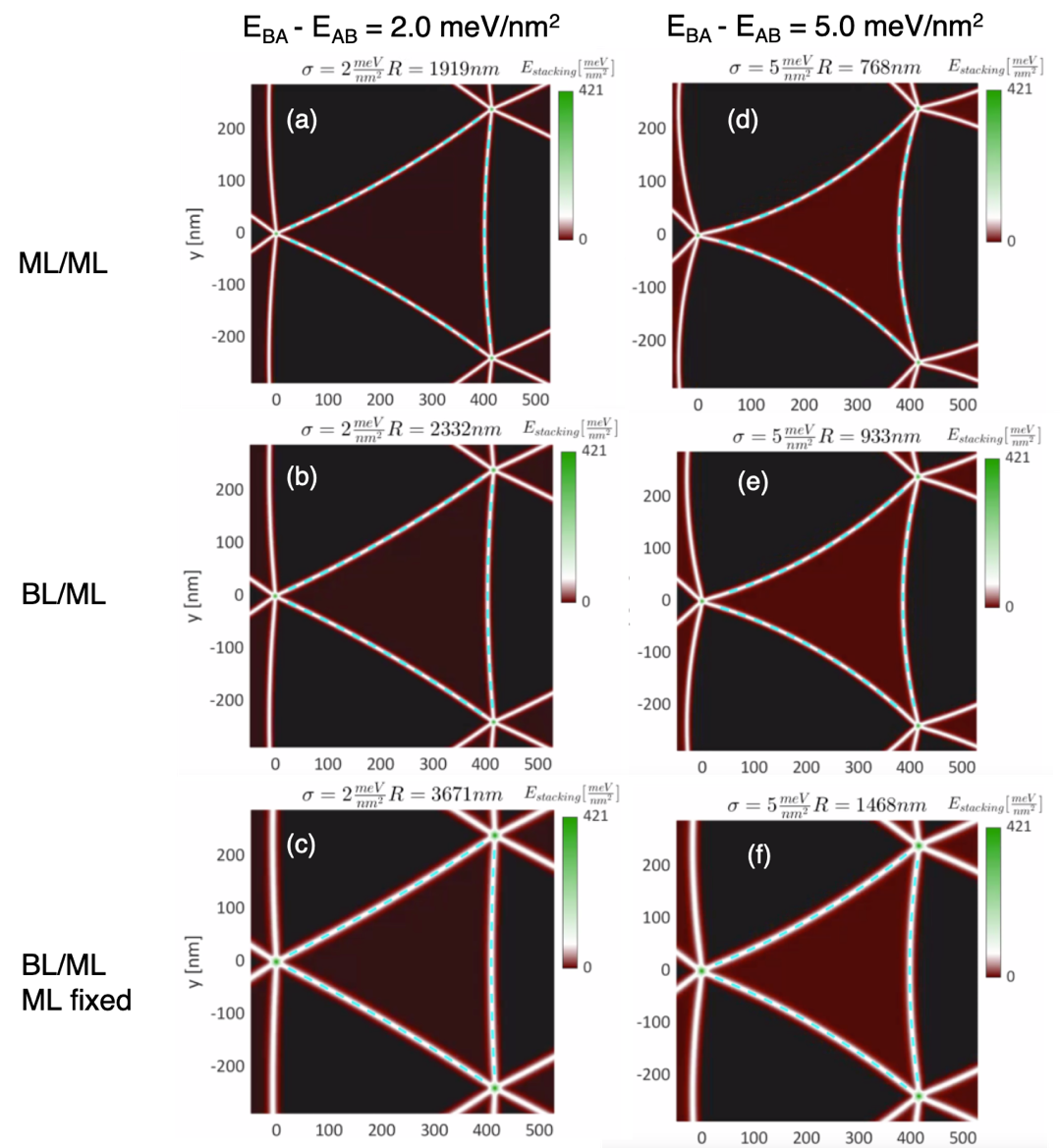


**Supplementary Figure 13: Relaxation calculations with added domain curvature.** Modified-GSFE relaxation calculations for (a) E_BA_ – E_AB_ = 2 meV/nm^2^ and monolayer/monolayer (ML/ML) conditions, (b) bilayer/monolayer (BL/ML) conditions, (c) BL/ML with a fixed bottom layer. (d-f) Same boundary conditions but with E_BA_ – E_AB_ = 5 meV nm^-2^.

**Supplementary Note** **10: t-hBN PFM response with various annealing conditions**

We find significant changes in domain curvature and AB/BA PFM contrast after annealing, shown for example in Supplementary Figure 14a and Supplementary Figure 14b. One explanation is thermally-driven lattice relaxation towards the energetically favorable *ab* stacking. Annealing-induced thermal drift in graphene/hBN heterostructures towards lattice alignment has been documented previously^17^. A prominent curvature is conveyed in the pre-500 ^o^C annealing image (a) by the double-sided arrow connecting two AA sites. Upon higher temperature annealing (b), the overall magnitude of the curvature appears to decrease, in agreement with our expectations of the *ab-*induced stacking-energy difference. By contrast, heating the sample above and below the (currently unknown) ferroelectric Curie temperature would not be expected to change the curvature. Still, *ab* stacking is surprising because we do not measure any hBN/graphite moiré patterns in these samples. Since relaxation effects can occur below the spatial resolution of PFM and nano-IR imaging, we may require higher-resolution scanning-probe techniques, e.g. scanning tunneling microscopy, to directly confirm *ab* alignment.


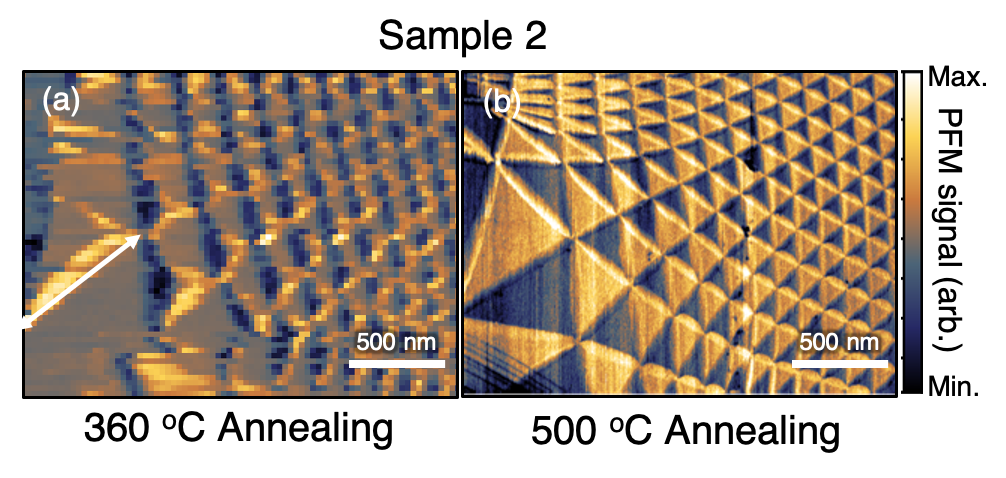


**Supplementary Figure 14**: **Sample 2 domain curvature at different annealing stages.** (a) PFM image of sample 2 after 360 ^o^C annealing. (b) PFM image of sample 2 after 500 ^o^C, reproduced from Fig. 1f of the main text

**Supplementary Note** **11: Effect of graphite substrate on ferroelectricity**

While our ab-initio calculations found no direct evidence of ferroelectric coupling to the phonons, we did obtain a stacking-dependent modification of the ferroelectric polarization $\Delta P$ in the presence of graphite. These dipole moments change monotonically with hBN/graphite separation (in a direction dependent on the stacking), but our calculations cannot comment whether they remain equal and opposite.


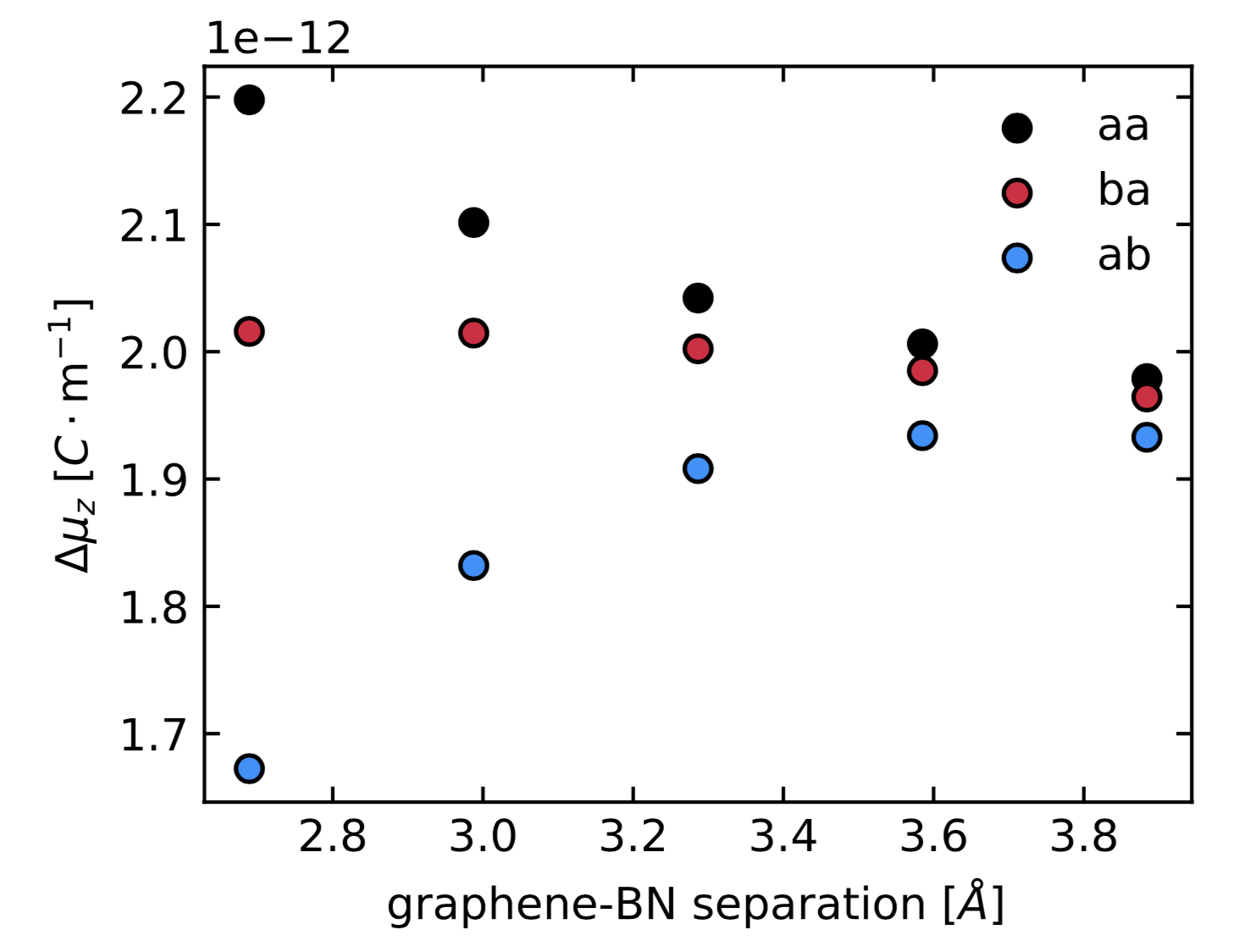


**Supplementary Figure 15: Tuning ferroelectricity with a graphene substrate.** Difference in polarization calculated in one unit cell of different stacking configurations of bilayer BN on graphene.

To gauge the role of ferroelectricity in the substrate-induced AB/BA shift, we calculated the same graphite-induced effect with AA’ (paraelectric) stacking. The result, Supplementary Figure 16, shows a similar level of frequency variation as its ferroelectric counterpart. This provides evidence that the observed AB/BA frequency shift does not require ferroelectricity in its origin.


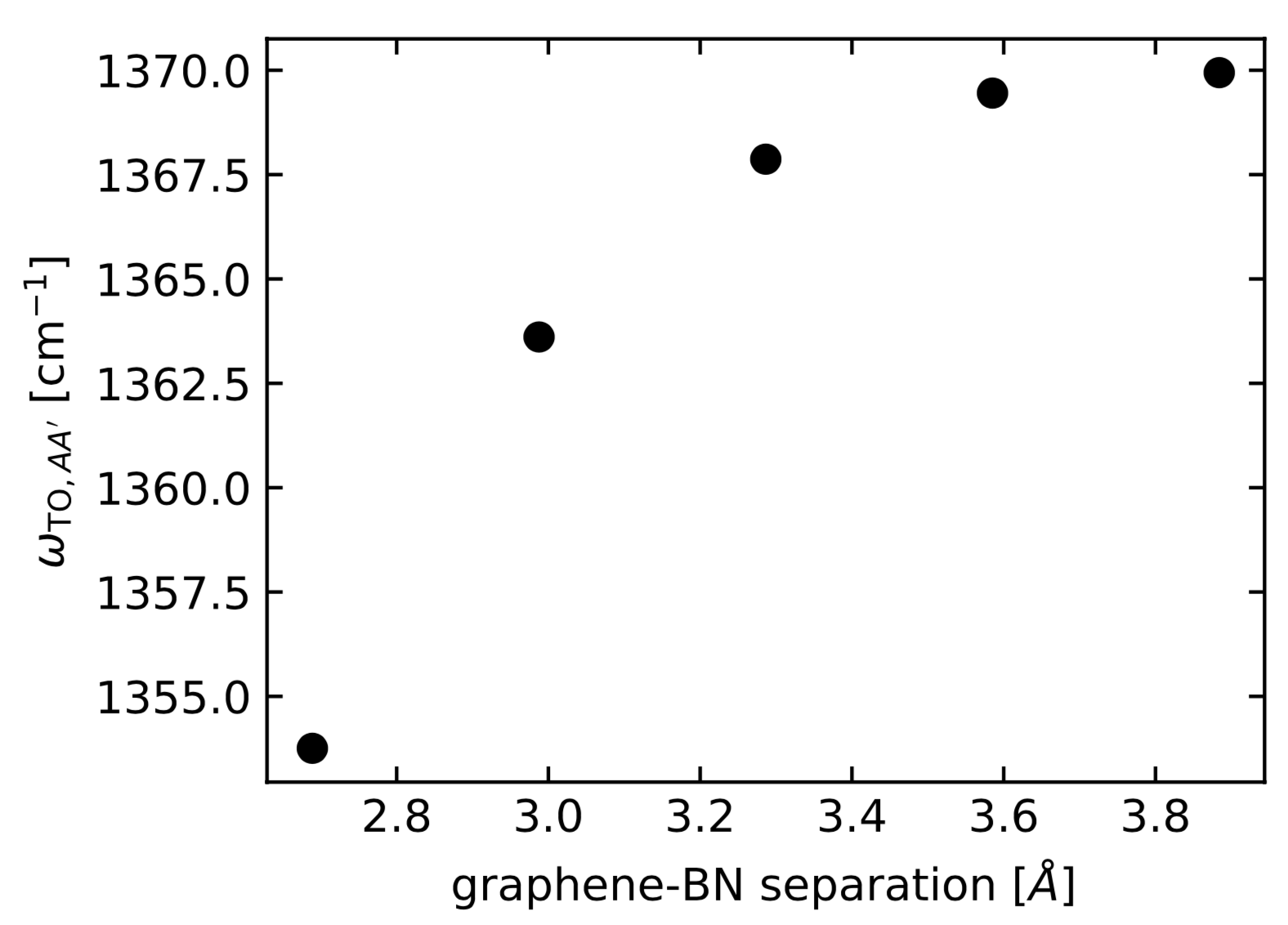


**Supplementary Figure 16:** **AA’-stacking TO frequency dependence on graphene separation**

**Supplementary Note** **12: PFM imaging on other substrates**

We find similar t-hBN moiré pattern shapes and curvature on other substrates. Near-field imaging, however, was unable to quantify the AB/BA frequency shift. In the context of bulk hBN, the sharp resonance in the bulk overwhelms the near-field contrast in the monolayer. While the contrast between monolayer and WSe_2_ is strong, it cannot be annealed up to 500 ^o^C (for optimal cleanliness) without causing chemical degradation. Graphite, meanwhile, offers greater chemical stability and a featureless optical response in the range of the hBN TO phonon.


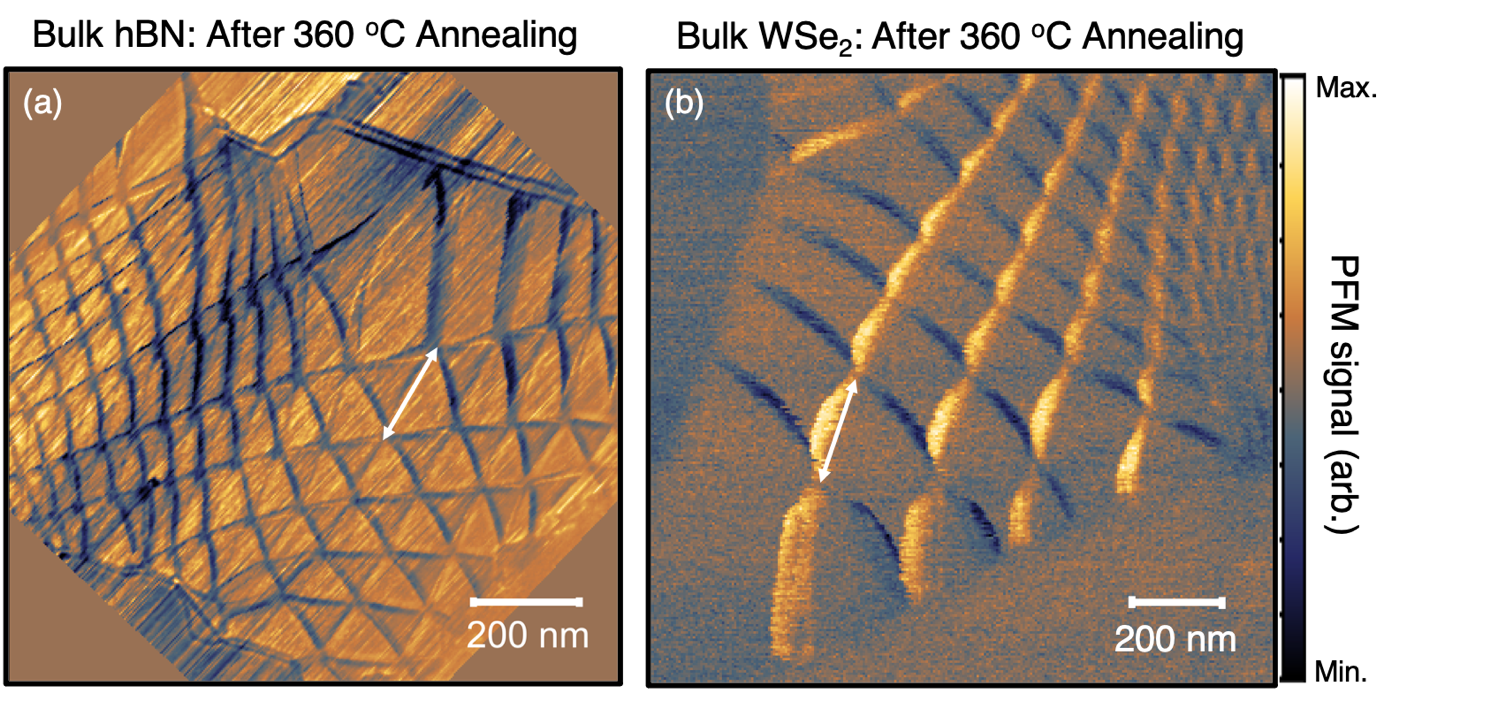


**Supplementary Figure 17: t-hBN moiré patterns on other substrates**. (a) bulk hBN and (b) bulk WSe_2_.

**Supplementary References**

1. Mcgilly, L. J. *et al.* Visualization of moiré superlattices. *Nat. Nanotechnol.* **15**, 580–584 (2020).

2. Yu, J. *et al.* Imaging Graphene Moiré Superlattices via Scanning Kelvin Probe Microscopy. *Nano Lett.* **21**, 3280–3286 (2021).

3. Woods, C. R. *et al.* Charge-polarized interfacial superlattices in marginally twisted hexagonal boron nitride. *Nat. Commun.* **12**, 347 (2021).

4. Yasuda, K., Wang, X., Watanabe, K., Taniguchi, T. & Jarillo-Herrero, P. Stacking-engineered ferroelectricity in bilayer boron nitride. *Science,* **372**, 1458–1462 (2021).

5. Vizner Stern, M. *et al.* Interfacial ferroelectricity by van der Waals sliding. *Science,* **372**, 1462–1466 (2021).

6. Gallagher, P. *et al.* Switchable friction enabled by nanoscale self-assembly on graphene. *Nat. Commun.* **7**, 10745 (2016).

7. Ni, G. X. *et al.* Soliton superlattices in twisted hexagonal boron nitride. *Nat. Commun.* **10**, 4360 (2019).

8. Constantinescu, G., Kuc, A. & Heine, T. Stacking in Bulk and Bilayer Hexagonal Boron Nitride. *Phys. Rev. Lett.* **111**, 036104 (2013).

9. Zhou, S., Han, J., Dai, S., Sun, J. & Srolovitz, D. J. Van der Waals bilayer energetics: Generalized stacking-fault energy of graphene, boron nitride, and graphene/boron nitride bilayers. *Phys. Rev. B* **92**, 155438 (2015).

10. Falin, A. *et al.* Mechanical properties of atomically thin boron nitride and the role of interlayer interactions. *Nat. Commun.* **8**, 15815 (2017).

11. Peng, Q., Ji, W. & De, S. Mechanical properties of the hexagonal boron nitride monolayer: Ab initio study. *Comput. Mater. Sci.* **56**, 11–17 (2012).

12. Halbertal, D. *et al.* Moiré metrology of energy landscapes in van der Waals heterostructures. *Nat. Commun.* **12**, 242 (2021).

13. Mohiuddin, T. M. G. *et al.* Uniaxial strain in graphene by Raman spectroscopy: G peak splitting, Grüneisen parameters, and sample orientation. *Phys. Rev. B - Condens. Matter Mater. Phys.* **79**, 205433 (2009).

14. Gorbachev, R. V. *et al.* Hunting for monolayer boron nitride: Optical and raman signatures. *Small* **7**, 465–468 (2011).

15. Dai, S. *et al.* Phonon Polaritons in Monolayers of Hexagonal Boron Nitride. *Adv. Mater.* **31**, 1806603 (2019).

16. Huang, S. *et al.* Low-Frequency Interlayer Raman Modes to Probe Interface of Twisted Bilayer MoS2. *Nano Lett.* **16**, 1435–1444 (2016).

17. Wang, L. *et al.* Evidence for a fractional fractal quantum Hall effect in graphene superlattices. *Science,* **350**, 1231–1234 (2015).
